# Extracellular RNAs from Whole Urine to Distinguish Prostate Cancer from Benign Prostatic Hyperplasia

**DOI:** 10.3390/ijms251810079

**Published:** 2024-09-19

**Authors:** Michele Stella, Giorgio Ivan Russo, Rosario Leonardi, Daniela Carcò, Giuseppe Gattuso, Luca Falzone, Carmen Ferrara, Angela Caponnetto, Rosalia Battaglia, Massimo Libra, Davide Barbagallo, Cinzia Di Pietro, Salvatore Pernagallo, Cristina Barbagallo, Marco Ragusa

**Affiliations:** 1 Department of Biomedical and Biotechnological Sciences, Section of Biology and Genetics “G. Sichel”, University of Catania, 95123 Catania, Italy; michele.stella@unict.it (M.S.); carmen.ferrara@phd.unict.it (C.F.); angela.caponnetto@unict.it (A.C.); rosalia.battaglia@unict.it (R.B.); dbarbaga@unict.it (D.B.); dipietro@unict.it (C.D.P.); mragusa@unict.it (M.R.); 2 Department of Urology, Polyclinic Hospital, University of Catania, 95123 Catania, Italy; giorgioivan.russo@unict.it; 3 Casa di Cura Musumeci GECAS, 95030 Gravina di Catania, Italy; leonardi.r@tiscali.it; 4 Department of Medicine and Surgery, University of Enna KORE, 94100 Enna, Italy; 5 Istituto Oncologico del Mediterraneo, 95029 Viagrande, Italy; daniela.carco@grupposamed.com; 6 Department of Biomedical and Biotechnological Sciences, Oncologic, Clinical and General Pathology Section, University of Catania, 95123 Catania, Italy; peppeg9305@gmail.com (G.G.); luca.falzone@unict.it (L.F.); mlibra@unict.it (M.L.); 7 DESTINA Genomica S.L., Health Sciences Technology Park (PTS), Av. de la Innovación 1, Building Business Innovation Center (BIC), 18016 Granada, Spain; salvatore.pernagallo@destinagenomics.com

**Keywords:** ncRNA, miRNA, lncRNA, diagnosis, liquid biopsy, molecular signature, PCa, BPH

## Abstract

RNAs, especially non-coding RNAs (ncRNAs), are crucial players in regulating cellular mechanisms due to their ability to interact with and regulate other molecules. Altered expression patterns of ncRNAs have been observed in prostate cancer (PCa), contributing to the disease’s initiation, progression, and treatment response. This study aimed to evaluate the ability of a specific set of RNAs, including long ncRNAs (lncRNAs), microRNAs (miRNAs), and mRNAs, to discriminate between PCa and the non-neoplastic condition benign prostatic hyperplasia (BPH). After selecting by literature mining the most relevant RNAs differentially expressed in biofluids from PCa patients, we evaluated their discriminatory power in samples of unfiltered urine from 50 PCa and 50 BPH patients using both real-time PCR and droplet digital PCR (ddPCR). Additionally, we also optimized a protocol for urine sample manipulation and RNA extraction. This two-way validation study allowed us to establish that miRNAs (i.e., miR-27b-3p, miR-574-3p, miR-30a-5p, and miR-125b-5p) are more efficient biomarkers for PCa compared to long RNAs (mRNAs and lncRNAs) (e.g., PCA3, PCAT18, and KLK3), as their dysregulation was consistently reported in the whole urine of patients with PCa compared to those with BPH in a statistically significant manner regardless of the quantification methodology performed. Moreover, a significant increase in diagnostic performance was observed when molecular signatures composed of different miRNAs were considered. Hence, the abovementioned circulating ncRNAs represent excellent potential non-invasive biomarkers in urine capable of effectively distinguishing individuals with PCa from those with BPH, potentially reducing cancer overdiagnosis.

## 1. Introduction

Prostate cancer (PCa) is a major health issue, with approximately 1.3 million new cases diagnosed worldwide every year. About 10 million men are presently living with a diagnosis of PCa, and approximately 700,000 of them are living with metastatic disease [1,2]. Despite recent advancements, PCa continues to wield a substantial impact on affected individuals, characterized by the paradox of excessive intervention for inherently non-malignant conditions (patients with very low-risk class) and insufficiencies in therapeutic approaches tailored to metastatic PCa.

Tumors are graded to allow better predictions for prognosis. For many years, the Gleason Grading System has been the most commonly used system, where cancers are scored according to the prostate tissue appearance under histological analysis. The tumor tissue is assigned a grade from 1 to 5 based on how closely it resembles normal prostate tissue. Two grades are assigned for the two most commonly observed tumor patterns to generate the Gleason score, and these scores are added together for a final Gleason sum. Gleason sums of 6, 7, and 8–10 represent low-, intermediate-, and high-grade cancer, respectively. In 2014, the International Society of Urological Pathology (ISUP) convened a panel of expert urologic pathologists and clinicians to address issues related to PCa grading [3], and the recommendations of the panel were adopted by the World Health Organization (WHO) in the 2016 classification of prostate tumors [4]. This new system grades the cancer between 1 and 5 depending on the Gleason score. The lower the grade, the less likely the tumor is to spread.

PCa diagnoses at mature stages of the disease and failure of therapy are the main factors leading to the increased mortality rate. To date, there is no single, specific test for PCa and it has conventionally been diagnosed by a combination of methods, including digital rectal examination (DRE), magnetic resonance imaging (MRI) scans, and prostate tissue biopsy, which remains, until now, the only exam that can confirm the presence of cancer and has the ability to provide a risk grade as well [5,6]. However, a biopsy can only capture the morphology of the tumor as a snapshot at a specific time and location and does not correspond to the whole characteristics or function of the tumor, which is highly heterogeneous. For this reason, even in the case of low-risk carcinoma, active surveillance is prescribed by multiple follow-up visits with imaging studies and repeated biopsies. This procedure significantly reduces patient compliance and quality of life, and it may be cost prohibitive [7]. In order to overcome these issues and capture tumor heterogeneity, a non-invasive method to monitor tumor-wide molecular scenario information during tumor progression or treatment responses is needed.

Liquid biopsy can be an answer to these challenges. Bodily fluids contain large amounts of substances secreted from cells as mediators of intercellular communication or released upon cell death. They include metabolites, proteins, and nucleic acids, which may reflect the pathophysiological status of the organism. Liquid biopsy refers to non-invasive sampling of bodily fluids to obtain tumor-derived materials, with the aim of measuring clinically informative cells, proteins, or nucleic acids detached from or secreted (actively or passively) by the tumor [8]. In this regard, the prostate-specific antigen (PSA) test remains the keystone for PCa screening [5,6]. The use of serum PSA has led to a significant increase in the number of diagnosed cases [9]. However, despite being the current gold standard test, evidence suggests that PSA lacks specificity [10]. PSA levels may be elevated even in the absence of PCa, and vice versa [11,12,13]. Among the other conditions altering PSA, benign prostatic hyperplasia (BPH) can be listed. For this reason, it is crucial to distinguish patients with BPH at an early stage to avoid unnecessary and invasive tests. PSA testing alone is insufficient for this differentiation. BPH is characterized by non-malignant prostate enlargement due to cellular hyperplasia in the transitional zone. This condition is strongly associated with aging, where the proliferation of prostatic cells leads to increased prostate size, urethral obstruction, and lower urinary tract symptoms (LUTS). Risk factors for BPH include advanced age, reduced testicular function, metabolic syndrome, family history of BPH, and obesity [14]. Although several studies have explored a potential link between BPH and PCa, the underlying pathophysiological mechanisms connecting the two conditions remain unclear [14,15]. 

Another disadvantage of PSA testing is that, though minimally invasive, it involves blood sampling. Therefore, there is a growing need to explore alternatives to PSA that offer better specificity and sensitivity and that can be detected in other biological fluids, such as urine. Urine is a biological fluid consisting of organic and inorganic compounds, including salts, cells (leucocytes, renal cells, urothelial cells, prostate cells, and exfoliated tumor cells), and tumor cell-free nucleic acids. Tumor-derived DNA, mRNAs, long non-coding RNAs (lncRNAs), and microRNAs (miRNAs) can be obtained via whole urine sampling, and circulating nucleic acids or cells (including those derived from the tumor) can be separately collected by centrifugation to obtain a urine cell pellet and a supernatant fraction [16]. Urine can be collected in large quantities, which addresses one of the major limitations of tissue biopsies and other bodily fluids, that often suffer from limited quantities. Additionally, urine collection is more patient friendly, as it can be performed anywhere, unlike the sampling of other bodily fluids or tissues, which must be collected in clinics or hospitals. Thus, urinary biomarkers offer a non-invasive and easily accessible means of assessing an individual’s health and susceptibility to PCa.

Several types of RNA are present and measurable in urine, such as lncRNAs, mRNAs, and miRNAs. There are abundant RNA hydrolases in urine, such as RNA-hydrolyzing enzyme (RNase II) and ribonuclease I, which hydrolyze both RNAs and DNA. Despite this mechanism, RNAs are still detectable in urine because they are somewhat protected from degradation by their encapsulation into extracellular vesicles (EVs), complexation in ribonucleoproteins, transportation in lipoproteins [17,18,19,20], or binding to RNA-binding proteins (RBPs), avoiding degradation by hydrolytic enzymes. These phenomena stabilize them to the point that they can withstand several cycles of freeze and thaw and remain stable at room temperature for long periods. RNA levels can be evaluated in different fractions, such as non-centrifuged urine, urine sediment, supernatant, and as part of EVs, such as exosomes [21]. RNAs represent a new source of reliable biomarkers that can be diagnostic, prognostic, and predictive during therapy of patients and have been widely studied in cancers of the urogenital system, including prostate [22,23,24,25].

This study aimed to evaluate the ability of a set of RNA urinary biomarkers, including lncRNAs, mRNAs, and miRNAs, to discriminate between PCa and BPH. The biomarkers were tested using real-time PCR and droplet digital PCR (ddPCR).

## 2. Results

### 2.1. Urine Manipulation Protocol Optimization

The optimization process encompassed several aspects: the selection of the starting sample (whole urine, centrifuged urine, or pellet); the initial volume for performing total RNA extraction; and the feasibility of utilizing devices to enhance the total RNA concentration within the sample. 

We examined various initial urine volumes for RNA extraction: 400 µL, 800 µL, 1500 µL, and 2000 µL. The total RNA yield ranged from approximately 60 ng (using 400 µL of urine) to 400 ng (using 2000 µL of urine). As expected, the highest RNA yields were achieved when utilizing whole urine (non-centrifuged), reaching up to 400 ng/µL of total RNA from 2000 µL of urine, particularly in samples obtained from BPH donors.

**Figure 1:**
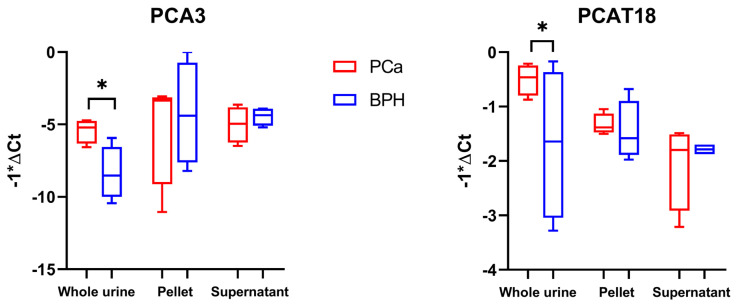
Expression levels of prostate cancer associated 3 (PCA3) and prostate cancer associated transcript 18 (PCAT18) in whole urine, pellet, and supernatant samples. Glyceraldehyde-3-phosphate dehydrogenase (GAPDH) was used as the endogenous control. A test set of 8 urine samples from prostate cancer (PCa) patients and 8 from benign prostatic hyperplasia (BPH) patients was used for the analyses. To determine the statistical significance, a *t*-test was applied (* = *p*-value < 0.05).

Through the expression analysis via real-time PCR of two lncRNAs from our biomarker list (presented in the materials and methods section of this paper and obtained by literature data mining), namely, prostate cancer associated 3 (PCA3) and prostate cancer associated transcript 18 (PCAT18), we evaluated which type of sample—whole urine (non-centrifuged), pellet, or supernatant (obtained after centrifugation)—was most suitable for our study. A test set of 8 urine samples from PCa patients and 8 from BPH patients was analyzed. For each patient, whole urine and the pellet and supernatant obtained after centrifugation were analyzed (average RNA yields extracted from fractions are shown in Supplementary Materials, Table S1). As depicted in Figure 1, PCA3 and PCAT18 were found to be significantly overexpressed in whole urine samples from PCa compared to BPH donors, confirming the upregulation trend reported in the literature. This was in contrast to observations in the pellet and supernatant samples, where overexpression was not confirmed and the distributions of the two pathological groups mostly overlapped with each other.

Performing real-time PCR on long RNAs extracted from urine is not an easy procedure due to the inherent dilution of all molecules circulating in the urine. This led to low RNA concentrations after extraction, making it difficult to conduct reliable and reproducible real-time PCR reactions. Therefore, we investigated a procedure involving urine sample concentration through a centrifugal concentrator, aiming to increase the sample starting volume using an easy, quick, and cost-effective procedure. We evaluated the expression levels of a set of RNA transcripts (PCAT18, glyceraldehyde-3-phosphate dehydrogenase [GAPDH], miR-16-5p, and miR-29c-3p) in non-concentrated (2 and 6 mL of urine, respectively) and concentrated (6 mL starting volume) samples and also in the waste fraction obtained after concentration to verify the eventual elution and loss of RNA molecules. Total RNA yields and urine starting volumes are shown in Table 1.

**Figure 2:**
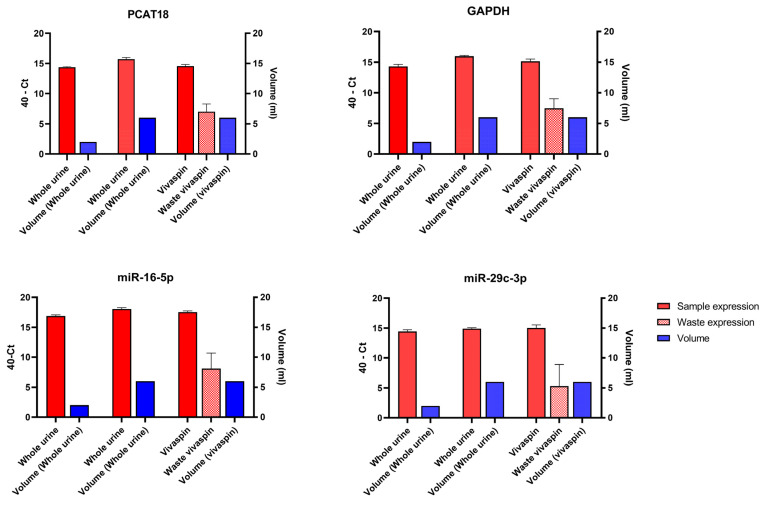
Expression levels of PCAT18, GAPDH, miR-16-5p, and miR-29c-3p in concentrated and non-concentrated urine samples. The graphs reveal a negligible gain in Ct values within the concentrated samples. The analysis was conducted on a set of 5 BPH urine samples. For each transcript, expression values are shown as 40-Ct in all tested samples, namely, whole and concentrated urine. For each sample, the starting volume is shown on the right y-axis. For the concentrated urine, the waste fraction was also analyzed to assess the eventual elution and loss of RNA molecules.

We tested the amplifiability of 1 µL of these RNA samples, revealing that despite starting with a urine sample volume three times greater (6 mL vs. 2 mL), the use of concentrator tubes did not result in a significant increase in signal detection. Specifically, evaluating the gain in terms of cycle threshold (Ct) through real-time PCR analysis showed a decrease of just 1 Ct for the specific transcripts used as tests (Figure 2). Moreover, the presence of an amplification signal in the waste fraction suggested some RNA molecules were lost during the concentration process.

### 2.2. RNA Expression in Urine from PCa vs. BPH 

**Figure 3:**
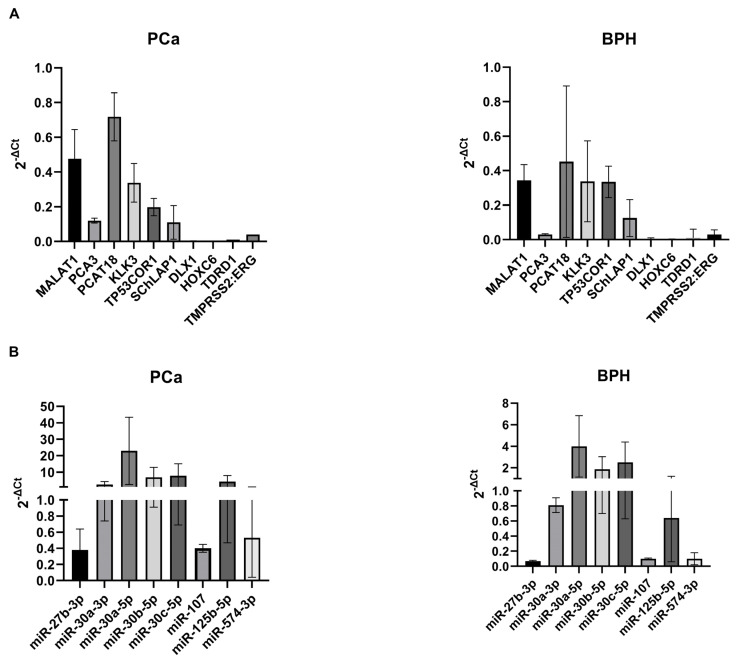
The graphs illustrate the mean 2^−ΔCt^ of selected long RNAs (**A**) and microRNAs (miRNAs) (**B**) in PCa and BPH whole urine samples. The threshold we set (cycle threshold [Ct] < 30) corresponds to a value of 2^−ΔCt^ < 0.1. A test set of 15 urine samples from PCa patients and 15 from BPH patients was used for the analyses.

The results of preliminary screening using real-time PCR on a sample test set (*n* = 15 PCa and *n* = 15 BPH) showed that some selected RNAs, although already positively tested in prostate and/or PCa cell line RNAs, were not easily amplifiable using 50 ng of RNA from whole urine (non-centrifuged): distal-less homeobox 1 (DLX1), ERG, homeobox C6 (HOXC6), SWI/SNF complex antagonist associated with prostate cancer 1 (SChLAP1), tudor domain containing 1 (TDRD1), and the fusion transcript TMPRSS:ERG showed Ct values undetermined or higher than 35 or presented dissociation curves with more than one peak, indicating low specificity of the amplified cDNA. In the latter case, additional primer pairs were designed, but a specific amplification was impossible to achieve. On the other hand, kallikrein related peptidase 3 (KLK3) (the mRNA coding for PSA), metastasis associated lung adenocarcinoma transcript 1 (MALAT1), PCA3, PCAT18, and tumor protein p53 pathway corepressor 1 (TP53COR1) showed Ct values < 30 using whole urine. Considering these results, we selected all those transcripts that had a Ct value < 30 in PCa samples (Figure 3A), dissociation curves with only one peak, and a melting temperature corresponding to the one expected for the specific amplicon.

**Figure 4:**
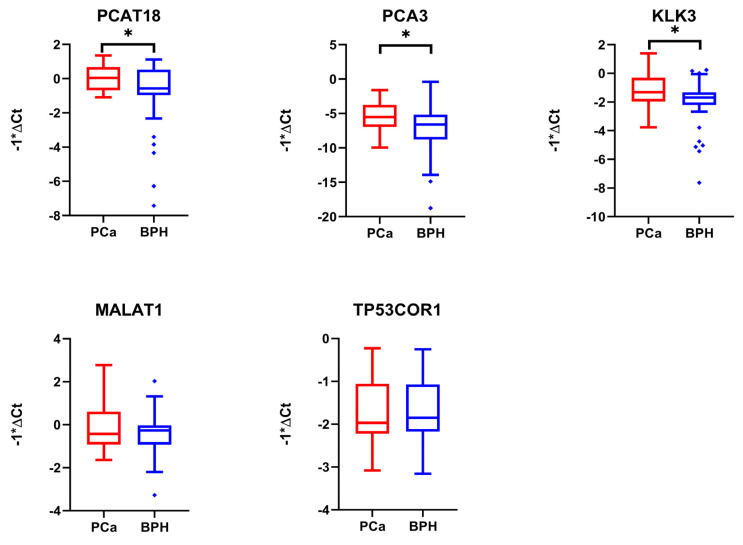
Box plots showing the expression levels of long non-coding RNAs (lncRNAs) and mRNAs selected and analyzed in a cohort of 50 PCa samples and 50 BPH samples used as controls. Y-axes report the value of ΔCt multiplied by −1. To determine the statistical significance, a *t*-test was applied (* = *p*-value < 0.05).

After this preliminary test, the expression of the selected lncRNA and mRNA putative biomarkers was evaluated through real-time PCR in a sample validation set composed of RNAs extracted from 50 PCa donors’ and 50 BPH donors’ whole urine samples. Real-time PCR analysis showed that PCAT18, PCA3, and KLK3 were significantly over-expressed in PCa samples compared to BPH samples, while MALAT1 and TP53COR1 showed no statistical significance (Figure 4 and Table 2).

### 2.3. miRNA Expression in Urine from PCa vs. BPH

**Figure 5:**
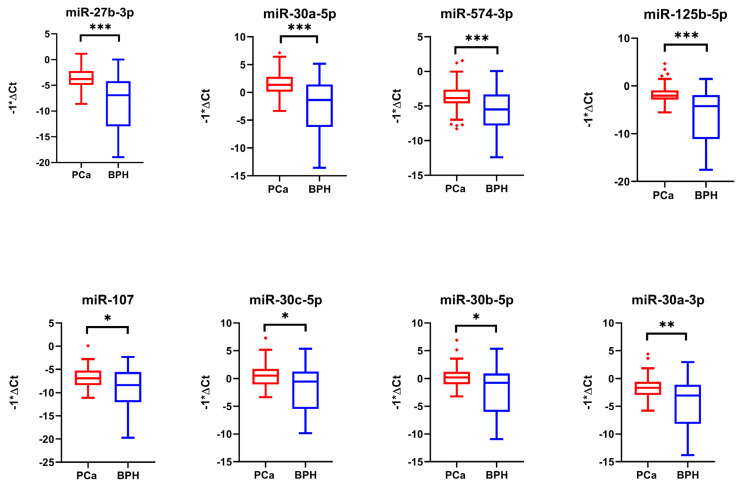
Box plots showing the expression levels of the miRNAs selected and analyzed in a cohort of 50 PCa samples and 50 BPH samples used as controls. Y-axes report the value of ΔCt multiplied by −1. To determine the statistical significance, a *t*-test was applied (* = *p*-value < 0.05, ** = *p*-value < 0.01, *** = *p*-value < 0.005).

A procedure similar to the one described for long transcripts was followed for miRNAs. As a preliminary step, we tested the expression levels of the selected miRNAs using TaqMan probes on the whole urine sample test set used for the long transcripts (*n* = 15 PCa and *n* = 15 BPH). The results showed that miRNAs were more easily amplifiable than long RNAs when 10 ng input RNA was used, showing a mean Ct value of 26.92 (StdDev ± 2.91) considering all miRNAs selected (Figure 3B). Therefore, the expression levels of all miRNA candidates were then analyzed on the validation sample set (*n* = 50 PCa and *n* = 50 BPH). The analysis results showed that miR-27b-3p, miR-30a-3p, miR-30a-5p, miR-30b-5p, miR-30c-5p, miR-107, miR-125b-5p, and miR-574-3p confirmed the overexpression in PCa samples compared to BPH samples, with relevant statistical significance (Figure 5 and Table 3).

### 2.4. Evaluation of the Diagnostic Accuracy of DE RNAs in Urine Using Data from Real-Time PCR 

**Figure 6:**
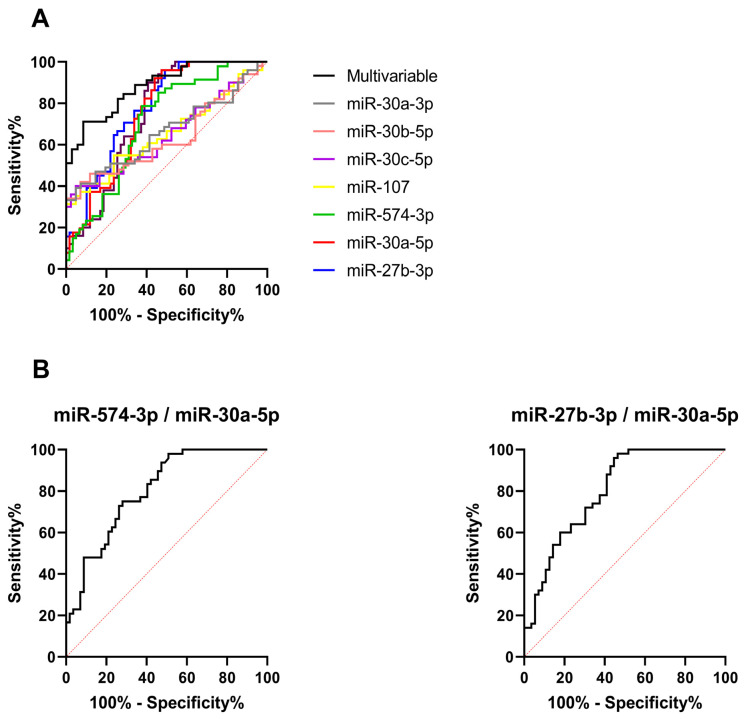
Receiver operating characteristic (ROC) curves of dysregulated miRNAs obtained by computing real-time PCR data. The area under the curve (AUC) value of each target is shown in Table 4. (**A**) Univariable and multivariable ROC curves; (**B**) ROC curves of miRNA molecular signatures.

**Figure 7:**
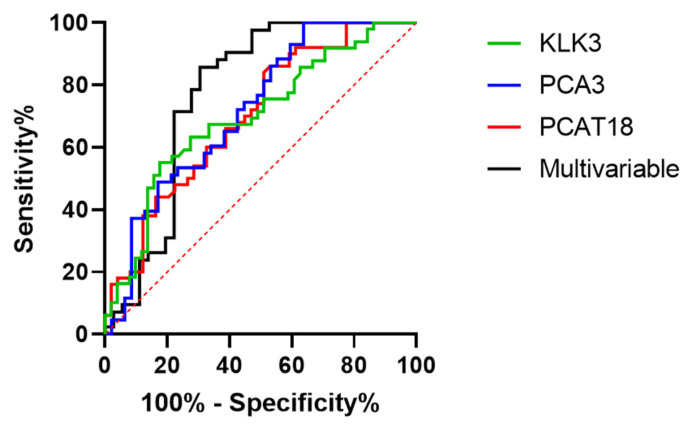
ROC curves of dysregulated lncRNAs and mRNAs obtained by computing real-time PCR data. The AUC of each target is shown in Table 5.

Data on lncRNAs, mRNAs, and miRNAs differentially expressed (DE) in the urine of PCa compared to BPH patients showed their potential application as tumor biomarkers. To assess their diagnostic accuracy, we computed receiver operating characteristic (ROC) curves using ΔCt values (Figure 6 and Figure 7). Specifically, we computed univariate ROC curves considering each RNA as a single biomarker; moreover, we combined various biomarkers to evaluate the best molecular signatures. Among the miRNAs, miR-27b-3p, miR-574-3p, miR-30a-5p, and miR-125b-5p showed fair performance, with area under the curve (AUC) values higher than 0.7. On the contrary, long transcripts gave lower AUC values, with only PCA3 overcoming the 0.7 threshold. Two multivariable ROC curves including all DE miRNAs or all DE long transcripts were computed, leading to an improved diagnostic performance for miRNAs (AUC = 0.881) and a slight increase in AUC value for long transcripts (AUC = 0.773). Considering the different associations, the pairs miR-27b-3p/miR-30a-5p and miR-574-3p/miR-30a-5p not only showed increases in AUC compared to individual biomarkers but also improvements in specificity (Figure 6 and Table 4).

**Table 4:** Receiver operating characteristic (ROC) curve analysis of dysregulated miRNAs obtained by computing real-time PCR data. CI—Confidence Interval; PPV—Positive Predicted Value; NPV—Negative Predicted Value.

miRNA Marker	Area	Std. Error	*p*-Value	95% CI	Cut-Off	Sensitivity	Specificity	Accuracy	PPV	NPV
miR-27b-3p	0.78	0.04	<0.0001	0.694–0.864	7.13	0.96	0.51	0.72	0.63	0.94
miR-574-3p	0.7	0.05	0.0003	0.607–0.802	4.75	0.79	0.62	0.69	0.62	0.79
miR-30a-5p	0.75	0.05	<0.0001	0.663–0.843	1.22	0.96	0.52	0.73	0.64	0.94
miR-125b-5p	0.75	0.047	<0.0001	0.656–0.840	3.46	0.90	0.59	0.73	0.65	0.87
miR-107	0.65	0.056	0.0104	0.544–0.765	11.20	1.00	0.31	0.62	0.54	1.00
miR-30c-5p	0.65	0.057	0.0158	0.534- 0.758	2.00	0.95	0.40	0.65	0.57	0.91
miR-30b-5p	0.63	0.058	0.0276	0.519–0.747	2.26	0.93	0.42	0.65	0.57	0.87
miR-30a-3p	0.66	0.056	0.0078	0.551–0.772	4.90	0.95	0.39	0.64	0.56	0.91
Multivariable	0.88	0.035	<0.0001	0.811–0.952	//	0.76	0.69	0.72	0.66	0.77
miR-27b-3p /miR-30a-5p	0.80	0.041	<0.0001	0.720–0.884	//	0.78	0.62	0.70	0.65	0.76
miR-574-3p/ miR-30a-5p	0.80	0.041	<0.0001	0.721–0.884	//	0.73	0.72	0.72	0.67	0.76

**Table 5:** ROC curve analysis of dysregulated lncRNAs and mRNAs obtained by computing real-time PCR data. CI—Confidence Interval; PPV—Positive Predicted Value; NPV—Negative Predicted Value.

RNA Marker	Area	Std. Error	*p*-Value	95% CI	Cut-Off	Sensitivity	Specificity	Accuracy	PPV	NPV
PCAT18	0.67	0.052	0.0006	0.596–0.801	0.74	0.84	0.49	0.66	0.63	0.75
PCA3	0.72	0.053	0.0004	0.612–0.823	8.08	1.00	0.36	0.66	0.59	1.00
KLK3	0.69	0.052	0.0007	0.594–0.800	1.33	0.55	0.82	0.69	0.75	0.65
Multivariable	0.77	0.059	<0.0001	0.657–0.888	//	0.74	0.82	0.77	0.88	0.64

### 2.5. ddPCR Validation

**Figure 8:**
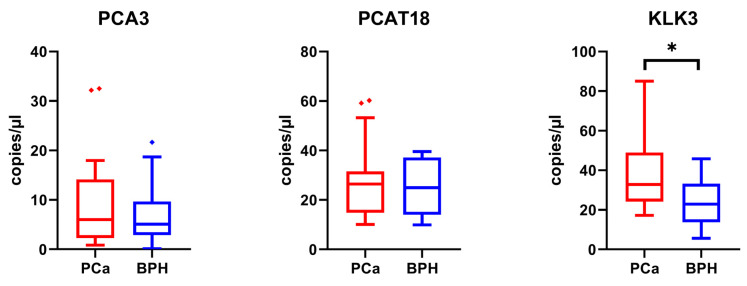
Box plots showing the expression levels of the lncRNAs and mRNAs selected and analyzed by ddPCR in a sub-cohort of 40 PCa samples and 40 BPH samples. Y-axes report the value of copies/µL. To determine the statistical significance, a *t*-test was applied (* = *p*-value <0.05).

We conducted further validation by analyzing the expression levels of the top transcripts (PCAT18, PCA3, and KLK3 as long RNAs, and 27b-3p, miR-30a-5p, miR-125b-5p, and miR-574-3p as miRNAs) in ddPCR using a sub-cohort of the validation sample set used for real-time PCR experiments, consisting of 40 PCa and 40 BPH samples. As depicted in the figure, the long RNAs PCAT18 and PCA3 were not found to be overexpressed in PCa as previously obtained by real-time PCR, while KLK3 upregulation was confirmed (Figure 8, Table 6).

**Figure 9:**
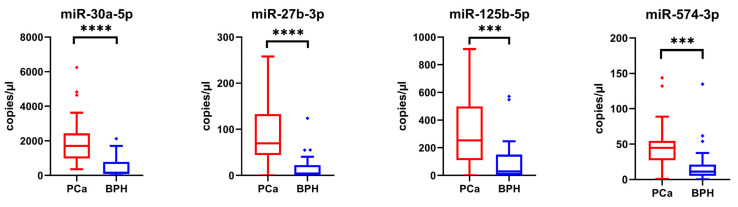
Box plots showing the expression levels of the miRNAs selected and analyzed by ddPCR in a sub-cohort of 40 PCa samples and 40 BPH samples used as controls. Y-axes report the value of copies/µL. To determine the statistical significance, a *t*-test was applied (*** = *p*-value < 0.001, **** = *p*-value < 0.0001).

Digital PCR analysis on the best-performing miRNAs (i.e., miR-27b-3p, miR-30a-5p, miR-125b-5p, and miR-574-3p) confirmed their statistically significant overexpression as previously observed through real-time PCR (Figure 9 and Table 7).

### 2.6. Evaluation of the Diagnostic Accuracy of the Best miRNA Biomarkers and Their Correlation with PSA and ISUP Using Data from ddPCR

**Figure 10:**
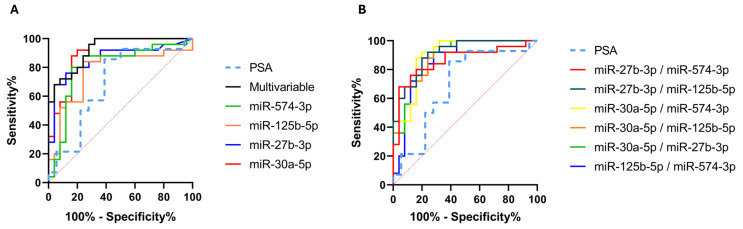
ROC curves generated using ddPCR data and prostate-specific antigen (PSA) values. The AUC of each target is shown in Table 8. (**A**) Univariable and multivariable ROC curves; (**B**) ROC curves of miRNA molecular signatures.

We utilized the miRNA biomarker expression data obtained from the ddPCR experiments to generate ROC curves (Figure 10). The newly generated curves highlighted the enhanced diagnostic capacity of these biomarkers, as evidenced by the values of AUC and various parameters of sensitivity, specificity, and accuracy shown in Table 8. The improvement in diagnostic capacity was also evident when analyzing the multivariable model, which presented an AUC equal to 0.92. Additionally, we combined various biomarkers to evaluate the optimal molecular signatures. All combinations demonstrated an AUC > 0.85 (Table 8). The ROC curves of various molecular signatures are shown in Figure 10, panel B. Notably, all miRNAs, considered both as single biomarkers or combined in pairs, showed better diagnostic performances than the PSA test (Figure 10 and Table 8).

**Figure 11:**
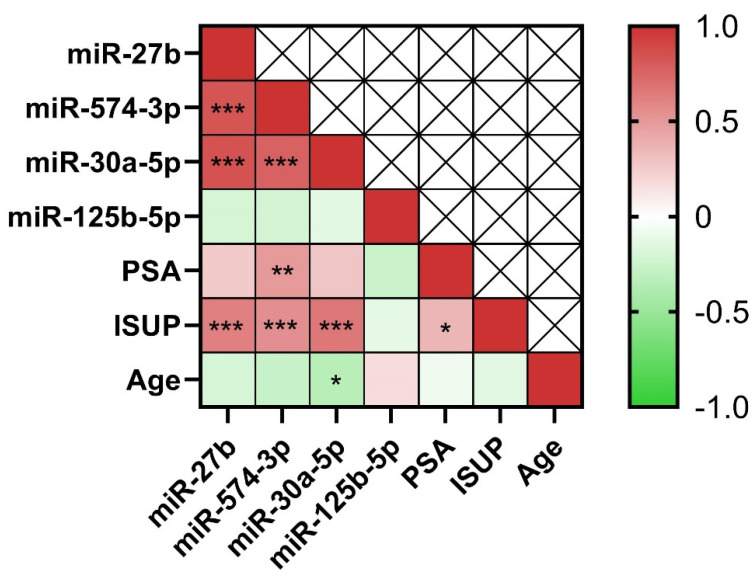
Heatmap showing correlations between the expression of miRNAs (expressed as counts/µL obtained by ddPCR) and clinicopathological data (PSA and International Society of Urological Pathology grade [ISUP] values). The scale bar shows *r*-values calculated through Spearman correlation tests, ranging from green (negative correlation) to red (positive correlation). Statistical significance is represented by the number of asterisks (* = *p*-value < 0.05; ** = *p*-value < 0.005; *** = *p*-value <0.0005).

Correlation analyses conducted using the ddPCR data revealed a pronounced positive and significant correlation between the expression levels of almost all tested miRNAs and the ISUP grade values; specifically, miR-27b-3p, miR-30a-5p, and miR-574-3p showed a *p*-value < 0.0005 (Figure 11). It was interesting to note a positive correlation between miR-574-3p copies/µL and PSA values. The same analysis was performed using real-time PCR data, and the results are shown in the Supplementary Materials (Figure S1).

## 3. Discussion

The role of ncRNAs in PCa holds crucial importance, contributing to the complexity of the molecular signaling involved in the pathogenesis of the disease. NcRNAs act as key regulators in cellular processes, influencing gene transcription, translation, and mRNA stability. Alterations in ncRNA expression in PCa can modulate tumor growth, metastasis, and therapeutic resistance [26]. Research on ncRNAs in PCa not only provides a more comprehensive overview of tumor biology but also lays a solid foundation for the development of personalized and targeted therapies, promoting a more effective approach to disease management. Integrating RNA biomarkers and liquid biopsy, a minimally invasive, more accurate, and comprehensive approach to PCa diagnosis and treatment can be achieved, especially using urine as a source of liquid biopsy.

We observed that the analysis on whole urine, as opposed to its fractions obtained after centrifugation (supernatant and pellet), provided a more comprehensive view of the dynamics of certain RNA molecules identified as PCa biomarkers. Indeed, some of these extracellular molecules could be freely circulating as naked RNAs or entrapped in protein complexes, vesicular structures, cellular debris, or detached cells, and centrifugation steps would lead to a loss of part of these potentially informative RNAs. In addition, the analysis of whole urine reduces the steps of sample processing and manipulation, which in turn facilitates its use as a liquid biopsy in clinical practice. To achieve an adequate final RNA concentration from urine, we found it more effective to use the extraction protocol of the Urine Cell-Free Circulating RNA Purification Kit (Norgen Biotek Corp., Thorold, Ontario, Canada) with respect to other column-based extraction kits from QIAGEN and Norgen (Supplementary Materials, Table S2). This protocol allowed the use of an initial urine volume of 2 mL. Also, at the end of the procedure, we added a precipitation step with sodium acetate and ice-cold 100% ethanol to further increase the concentration in a lower water volume of resuspension. Attempts to use concentrator tubes were less efficient than expected in terms of gain of Ct. We concluded that this minimal gain in signal detection does not justify the addition of a concentration step in the analysis. Moreover, from the perspective of the clinical application of RNA biomarkers, a simpler procedure would be more easily applied in clinical laboratories. Lastly, considering that it is advisable to minimize sample handling, the concentration step would only have been helpful if it had resulted in a significantly greater increase in the detected signal. Moreover, our data on wasted samples suggested that a small part of long and short RNAs could pass through the mesh of the filter of the Vivaspin concentrator tube, causing the loss of potentially informative biological material.

The selection of RNA biomarkers (lncRNAs, mRNAs, and miRNAs) for PCa was carried out by examining literature data that suggested the dysregulation of specific transcripts in the biofluids of patients with PCa compared to those of unaffected individuals. We adopted this approach to identify, validate, and propose the best biomarkers in unfiltered urine, not only based on their dysregulation but also on their diagnostic performance, and their discrimination ability between PCa- and BPH-affected individuals, a significant focus in contemporary diagnostic research. The identification of new biomarkers for PCa is of paramount importance compared to serum PSA, due to the lower diagnostic specificity and sensitivity of the latter. PSA levels can be elevated in conditions other than PCa, such as BPH, leading to potential overdiagnosis and unnecessary treatments [6,9,27]. The exploitation of multiple RNA biomarkers in urine offers a more dynamic method for distinguishing between PCa and BPH; this distinction is critical for improving diagnostic accuracy and ensuring that patients receive the most appropriate care.

The use of both real-time PCR and ddPCR for RNA analysis was critical because each technique offers unique advantages. Real-time PCR provides rapid and sensitive quantification of RNA expression levels, but its results are strongly linked to the use of an appropriate reference gene, calibrator sample, or, in the case of absolute quantification analysis, a standard curve. ddPCR offers good precision in quantifying rare RNA molecules, and its droplet-based partitioning allows for absolute quantification of target RNA molecules without the need for standard curves or endogenous controls [28,29,30,31]. The combination of these methods ensured the robust validation and high reproducibility of our results, underscoring the reliability of the identified RNA biomarkers. We chose to analyze biomarker expression using real-time PCR as the primary technique for its high sensitivity, ease of use, and low cost, in addition to being the most commonly used method for biomarker discovery in laboratories. Furthermore, unlike ddPCR, real-time PCR is more widely available in clinical laboratories, making it the gold standard technique for RNA-based biomarker applications in clinical practice. We selected ddPCR as the validation technique because it is reported to be even more sensitive and precise [30,31,32]. Given that ddPCR instruments are not available in all clinical laboratories to date, using ddPCR for validation allowed us to focus only on the strongest biomarkers, which can also be measured with real-time PCR in clinical practice. ddPCR allowed us to discard biomarkers with the poorest diagnostic performance, resulting in a list of reliable biomarkers for PCa.

In our study, real-time PCR was employed at first to validate as PCa biomarkers 5 long RNAs (MALAT1, PCA3, PCAT18, KLK3, and TP53COR1) and 8 miRNAs (miR-27b-3p, miR-30a-3p, miR-30a-5p, miR-30b-5p, miR-30c-5p, miR-107, miR-125b-5p, and miR-574-3p) that were reported to be differentially expressed in different biofluids and EVs from PCa patients. Subsequently, through a second validation step by ddPCR, we assessed the reproducibility of the statistically significant results previously obtained. Our analyses revealed that miRNAs have excellent potential as PCa biomarkers detectable in urine, capable of distinguishing individuals affected by PCa from those affected by BPH, by applying both PCR methods. Conversely, long RNAs did not prove to be fairly effective; in fact, their modest statistically significant dysregulations observed through real-rime PCR were lost when using ddPCR. This outcome may be explained by the differing nature of the molecules: miRNAs are remarkably stable in various bodily fluids, and they are resistant to degradation by RNases. This stability is attributed to their small size and their association with protective carriers like exosomes, microvesicles, and protein complexes [33]. Furthermore, they constitute the most prevalent RNA species present in bodily fluids, along with tRNA-derived molecules/fragments/transcripts [34,35]. These properties of miRNAs make them ideal candidates for biomarkers in clinical diagnostics and prognostics. On the other hand, extracellular long RNAs tend to be more susceptible to degradation by ribonucleases due to their larger size, and, although they can be found encapsulated within EVs, their concentration in biological fluids remains rather low [36]. The low stability and scarcity in the extracellular environment of long RNAs make it tricky to effectively apply their dosage for diagnostic purposes. This could explain why our results on long RNAs differed from those described in the literature, aside from the fact that our data came from non-filtered urine analysis, whereas the previously reported data were obtained from different fractions of urine or blood.

To explore the potential use of these transcripts as diagnostic urinary biomarkers, we assessed their diagnostic performance by generating ROC curves. Traditionally, single biomarkers have been used in diagnostics. However, single biomarkers can sometimes provide limited information due to biological variability, lack of specificity, or insufficient sensitivity. Multiple biomarkers refer to the use of more than one biomarker in combination, providing a molecular signature that enhances diagnostic precision. It is well established that a signature incorporating multiple biomarkers can exhibit higher sensitivity and specificity than a single biomarker. Accordingly, we created two types of ROC curves to identify the most effective biomarkers or combination of biomarkers, aiming to develop a specific signature for diagnosing PCa. Considering a single biomarker, the AUC ranged from 0.77 to 0.9. A significant increase in diagnostic test performance was observed when considering the molecular signature composed of 4 tested miRNAs (multivariable AUC = 0.92) as well as the molecular signatures composed of two miRNAs, where each of the tested pairs reported an AUC > 0.85. Additionally, each individual biomarker demonstrated better diagnostic performance compared to PSA. Finally, miR-27b-3p, miR-574-3p, miR-30a-5p, and miR-125b-5p emerged as the best biomarkers from our validation analysis and are overexpressed in the urine of PCa patients.

## 4. Materials and Methods

### 4.1. Patient Recruitment and Sample Cohorts

This study was conducted according to the Declaration of Helsinki and was approved by the ethics committee “Catania 2” (Protocol Number 173/C.E.; minutes n. 89/2022/CECT2). Written informed consent was obtained from each study participant. A total of 50 patients with PCa and 50 patients with BPH were recruited by the Istituto Oncologico del Mediterraneo (IOM) and the Azienda Ospedaliero-Universitaria Policlinico “G. Rodolico − S Marco” Catania.

Participants enrolled in this study were selected using the following criteria: PCa donors should not have other severe systemic diseases, such as kidney failure, diabetes, or neurodegenerative diseases; BPH patients should not have familiarity with PCa, an ongoing neoplastic pathology, or other serious systemic pathologies such as renal failure, diabetes, or neurodegenerative diseases. The groups of patients ranged in age between 50 and 75 years (age range of onset of PCa). Based on this sampling, a power (1-β error probability) of 0.80 was obtained by performing *t*-tests for two independent samples with an alpha error of 0.05 and an effect size (d) of 0.50. No healthy controls were enrolled in this study, as in the age range of 50–75 years it could be very difficult to identify an adequate number of people without medical problems with the prostate. Furthermore, the use of BPH patients as controls was considered diagnostically functional in our study, as BPH and PCa share part of the symptomatology and sometimes PSA levels are also elevated in BPH. Therefore, the identification of RNA markers capable of discriminating between the two somewhat similar conditions would be clinically more useful. Urine samples from cancer-unaffected male donors were used in the preliminary stages of testing. The means of age and clinicopathological parameters of the whole cohort are summarized in Table 9. All diagnoses of PCa were determined by histologic analysis of biopsy.

We performed preliminary analyses on a smaller sub-cohort, specifically the “samples test set” in the present manuscript. This set consisted of 15 urine samples from PCa donors and 15 urine samples from BPH donors. The same cohort was then expanded to comprehensively include 50 PCa donors’ urine and 50 BPH donors’ urine in order to confirm the findings in a larger sample set, named the “validation data set” in this article. Forty urine samples from PCa donors and 40 urine samples from BPH donors made up the sub-cohort of the validation sample set that was used for the ddPCR investigations.

### 4.2. Urine Collection and Sample Processing

Urine (first-morning urination) was collected in sterile disposable containers by the individuals recruited for the study. The processing of the urine was performed no later than two hours after the time of sample collection. For each patient, 12 mL of non-centrifuged urine (whole urine) was collected and stored at −80 °C. Moreover, an aliquot of urine (12 mL) from the same donor was centrifuged in 15 mL tubes for 10 min at 2000× *g* and 4 °C; the supernatant was recovered and collected in a new sterile tube and immediately frozen at −80 °C. For all samples, the pellets (containing cellular debris floating in the urine) obtained from centrifugation were also stored at −80 °C.

### 4.3. Urine Concentration

The total RNA contained in urine was concentrated using Vivaspin^®^ 6 concentrator tubes (Sartorius AG). Vivaspin^®^ 6 concentrator tubes are designed to concentrate molecules of interest through the process of ultrafiltration, ensuring efficient processing of samples from 2 to 6 mL. The device features two double vertical membranes with a 100 KDa molecular weight cut-off (MWCO) filter and a low-volume stop pocket that allows high processing speeds and maximum concentration factors. Six mL of whole urine was concentrated using these concentrator tubes by centrifugation for 15 min at 4000× *g* and 4 °C on a Beckman J-6M/E centrifuge (rotor JS 5.2). The resulting concentrate was collected and diluted in PBS to 2 mL for subsequent RNA extraction. For these tests, we analyzed the expression levels of one of the putative RNA biomarkers, namely PCAT18, and the potential endogenous controls GAPDH, miR-16-5p, and miR-29c-3p.

### 4.4. RNA Extraction from Urine Samples

RNA extraction was performed on 2 mL or 6 mL of whole urine following the protocol of Norgen’s Urine Cell-Free Circulating RNA Purification Kit (Norgen Biotek Corp.). Separately, RNA contained in centrifuged urine (supernatant) was extracted using the same protocol. Total RNA extracted was precipitated with sodium acetate, glycogen, and ice-cold absolute ethanol [37] to increase the concentration in a smaller volume of solvent. Total RNA was extracted from the urine pellet with TriZol^®^ (Thermo Fisher Scientific, Waltham, MA, USA), according to the manufacturer’s instructions. RNA was quantified and its quality was evaluated using a NanoDrop One spectrophotometer (Thermo Fisher Scientific) and Qubit fluorescence quantification system (Thermo Fisher Scientific), and then stored at −80 °C until the time of PCR analysis.

### 4.5. Transcript Selection and PCR Primer Design

By literature data mining, we selected for our study a set of RNA transcripts (lncRNAs, mRNAs, and miRNAs) involved in PCa and differentially expressed both at intracellular and extracellular levels, especially those dysregulated in the biological fluids (e.g., serum, urine, plasma, and EVs) of PCa patients (Table 10).

To analyze the expression of selected long RNA transcripts, we designed PCR primers. In addition, we designed specific primers for the housekeeping gene used for normalization, namely GAPDH (Table 11). Primer design was performed using the online tool PrimerBlast (http://www.ncbi.nlm.nih.gov/tools/primer-blast, last accessed on 9 August 2024).

All primers were first tested on commercial human prostate RNA (Ambion^®^) and PCa cell line RNAs (PC3, DU145, or LNCaP). The expression of selected lncRNAs and mRNAs in urine from PCa and BPH was investigated through real-time PCR using the Power SYBR Green RNA-to-CT™ 1-Step Kit (Thermo Fisher Scientific) with 20 ng of total RNA per reaction, according to the manufacturer’s instructions. With regard to miRNA analyses, miRNA-specific cDNA was reverse-transcribed using the TaqMan™ MicroRNA Reverse Transcription Kit (Thermo Fisher Scientific) from 10 ng of total RNA and amplified using TaqMan™ Universal Master Mix II, no UNG (Thermo Fisher Scientific) with TaqMan™ microRNA single assays for miR-27b-3p (ID 000409), miR-30a-3p (ID 000416), miR-30a-5p (ID 000417), miR-30b-5p (ID 000602), miR-30c-5p (ID 000419), miR-107 (ID 000443), miR-125b-5p (ID 000449), miR-574-3p (ID 002349), miR-16-5p (ID 000391), miR-29c-3p (ID 000587), and U6 (ID 001973). For each real-time PCR reaction, 5 ng of miRNA-specific cDNA was used. All real-time PCR reactions were performed on a 7900HT Fast Real-Time PCR System (Thermo Fisher Scientific). The results were analyzed through SDS RQ Manager 1.2 software (Thermo Fisher Scientific); normalization was performed using GAPDH (for lncRNAs and mRNAs) and U6 (for miRNAs). Expression fold changes of DE RNAs were calculated by applying the 2^−ΔΔCt^ method. Statistical analysis was performed by evaluating normality and homogeneity of variance of ΔCt distributions with GraphPad Prism 8 software. According to the results, a parametric or non-parametric *t*-test was applied. Statistical significance was established at a *p*-value < 0.05.

### 4.6. ROC Curve Analysis

To evaluate the diagnostic accuracy of RNA biomarkers dysregulated in PCa patient urine, ROC curves were computed through the software IBM SPSS Statistics v23 and GraphPad Prism v8. The AUC and 95% confidence interval (95% CI) were calculated to assess the accuracy of sensitivity and specificity parameters and to find an appropriate cut-off point. Statistical significance was established at a *p*-value < 0.05. Multivariable and univariable ROC curves were generated. For multivariable curves, binary logistic regression models were built and predicted values were used as input data for ROC curve computation. Sensitivity, specificity, accuracy, positive predictive value (PPV), and negative predictive value (NPV) were calculated using confusion matrixes, and predicted grouping was generated by the regression model.

### 4.7. ddPCR

We investigated the expression of lncRNAs and mRNAs using the QX200™ ddPCR™ EvaGreen Supermix Kit (Bio-Rad Laboratories, Inc., Hercules, CA, USA), while ddPCR™ Supermix for Probes™ (Bio-Rad Laboratories, Inc.) was used for miRNAs. cDNA was obtained using SuperScript™ III Reverse Transcriptase (Thermo Fisher Scientific), according to the manufacturer’s protocol. Total cDNA was retro-transcribed from 100 ng of RNA; 20 ng of cDNA was used for all ddPCR reactions. miRNAs were retro-transcribed using the TaqMan™ MicroRNA Reverse Transcription Kit, as reported above, and for each ddPCR reaction, 5 ng of miRNA-specific cDNA was used. Absolute quantification was performed.

The QX200 Droplets Generator was able to fractionate a mix of each cDNA sample, 17 µL of QX200™ ddPCR™ EvaGreen Supermix or ddPCR™ Supermix for Probes™, and 70 µL of Droplet Digital™ PCR Oil into approximately 20,000 droplets. Target amplification occurred in each droplet using the following thermal cycle: polymerase activation at 95 °C for 10 min, 40 cycles of amplification at 94 °C for 30 s (denaturation), 60 °C for 1 min (annealing/elongation), droplet stabilization at 98 °C for 10 min, followed by an infinite hold at 4 °C. A ramp rate of 2 °C/second was used between the steps of the amplification process. Data analysis was performed using QuantaSoft™ Analisys Pro Software V1.0 (Bio-Rad Laboratories, Inc.). Statistical analysis was performed by evaluating normality and homogeneity of variance of count distributions with GraphPad Prism 8 software. According to the results, a parametric or non-parametric *t*-test was applied. Statistical significance was established at a *p*-value < 0.05.

### 4.8. RNA Expression and Clinicopathological Parameters Correlation

The existence of a correlation between RNA expression and clinicopathological parameters was investigated. For quantitative variables, the correlation was evaluated by calculating the Pearson or Spearman correlation coefficient, according to the normality of expression value distributions, using copies/µL values and clinicopathological parameters as input data. The analysis was performed specifically on DE RNAs showing the best AUC.

## 5. Conclusions

In conclusion, our study validated the optimal shortlist of biomarkers for PCa (miR-27b-3p, miR-574-3p, miR-30a-5p, and miR-125b-5p) detectable in whole urine through the use of real-time PCR and ddPCR techniques. Given their resistance and stability, miRNAs have proven to be excellent biomarkers capable of effectively distinguishing individuals with PCa from those with BPH, potentially reducing cancer overdiagnosis. Furthermore, an enhancement in biomarker diagnostic performance was observed by analyzing the ROC curves of potential molecular signatures considering two or more miRNAs.

As a final consideration, it is important to note that RNA-based biomarkers are gaining attention as promising diagnostic tools for various diseases, including PCa. Their unique molecular characteristics enable more precise detection of disease states, potentially offering advantages over traditional biomarkers. However, the effectiveness of RNA-based biomarkers must be validated through further research, especially in larger cohorts including patients from different parts of the world. This is crucial due to the inherent heterogeneity of PCa, which can vary significantly among different demographic groups. In our study, RNA-based biomarkers showed superior diagnostic accuracy compared to the PSA test, which is currently the standard tool for PCa screening. This finding is significant as the PSA test has limitations, including lack of specificity and a high rate of false positives, leading to unnecessary procedures and overdiagnosis. The improved performance of RNA-based biomarkers suggests that they could either be used together with the PSA test to provide a more comprehensive diagnostic approach or, eventually, replace it. Before these biomarkers can be widely implemented, further validation in expanded cohorts will be necessary. Such studies will help to confirm their reliability across diverse populations, ensuring that the benefits observed in initial studies are consistent across broader clinical settings. If proven effective, RNA-based biomarkers could revolutionize the diagnosis of PCa, leading to more accurate detection and ultimately improving patient outcomes.

## Figures and Tables

**Table 1:** Total RNA yields and sample starting volumes of urine used to test the concentrator tubes.

Sample	Starting Volume	Mean RNA Yield
	(mL)	(ng) ± StdDev
Whole urine	2	190 ± 38.1
Whole urine	6	448 ± 76.16
Concentrate Urine Vivaspin 6	6	376 ± 45.5
Waste Urine Vivaspin 6	2	72 ± 11.52

**Table 2:** The table shows the *p*-values and fold change values for each RNA analyzed.

Gene	RNA Typology	*p*-Value	Fold Change
MALAT1	lncRNA	0.71	−1.03
PCA3	lncRNA	0.011	1.55
PCAT18	lncRNA	0.007	1.57
KLK3	mRNA	0.017	1.29
TP53COR1	lncRNA	0.71	−1.21

**Table 3:** The table shows the *p*-values and fold change values for each miRNA analyzed.

miRNA	*p*-Value	Fold Change
miR-27b-3p	<0.0001	22.22
miR-574-3p	<0.0001	2.65
miR-30a-5p	<0.0001	14.35
miR-125b-5p	<0.0001	24.42
miR-107	0.009	5.81
miR-30c-5p	0.015	5.22
miR-30b-5p	0.027	5.55
miR-30a-3p	0.007	7.68

**Table 6:** The table shows the *p*-values and ratio of PCa copies/BPH copies values for each long RNA analyzed.

RNA	Typology	*p*-Value	Median PCa/BPH
PCA3	lncRNA	0.771	1.18
PCAT18	lncRNA	0.853	0.98
KLK3	mRNA	0.038	1.43

**Table 7:** The table shows the *p*-values and ratio of PCa copies/BPH copies values for each miRNA analyzed.

miRNA	*p*-Value	PCa/BPH
miR-27b-3p	<0.0001	5.45
miR-30a-5p	<0.0001	4.34
miR-125b-5p	0.0001	3.20
miR-574-3p	0.0006	2.42

**Table 8:** ROC curve analysis of prostate-specific antigen (PSA) and dysregulated miRNAs obtained by computing ddPCR data.

miRNA ID	Area	Std. Error	*p*-Value	95% CI	Cut-Off	Sensitivity	Specificity	Accuracy	PPV	NPV
miR-30a-5p	0.9	0.043	<0.0001	0.819–0.989	911	0.88	0.84	0.86	0.85	0.88
miR-27b-3p	0.86	0.056	<0.0001	0.748–0.969	44.45	0.76	0.88	0.82	0.86	0.79
miR-125b-5p	0.77	0.071	0.0009	0.634–0.914	88.65	0.84	0.76	0.8	0.78	0.83
miR-574-3p	0.81	0.067	0.0002	0.672–0.937	19.06	0.88	0.76	0.82	0.79	0.86
Multivariable	0.92	0.034	<0.0001	0.854–0.991	//	0.84	0.76	0.8	0.78	0.83
miR-30a-5p /miR-27b-3p	0.89	0.045	<0.0001	0.807–0.984	//	0.8	0.84	0.82	0.83	0.81
miR-30a-5p /miR-125b-5p	0.91	0.041	<0.0001	0.823–0.984	//	0.72	0.8	0.76	0.78	0.74
miR-30a-5p /miR-574-3p	0.91	0.043	<0.0001	0.816–0.988	//	0.72	0.84	0.78	0.82	0.75
miR-27b-3p /miR-125b-5p	0.91	0.039	<0.0001	0.833–0.987	//	0.88	0.8	0.84	0.81	0.87
miR-27b-3p /miR-574-3p	0.86	0.054	<0.0001	0.754–0.97	//	0.76	0.88	0.82	0.86	0.79
miR-125b-3p /miR-574-3p	0.86	0.054	<0.0001	0.757–0.97	//	0.76	0.84	0.8	0.83	0.78
PSA	0.7	0.096	0.062	0.505–0.883	3.85	0.86	0.61	0.71	0.63	0.85

**Table 9:** Demographic data of the case and control cohorts involved in the study. ISUP: International Society of Urological Pathology grade.

Samples	*n*° of Samples	Mean Age	Mean PSA	Mean ISUP
		(Years) ± StdDev	(ng/mL) ± StdDev	(Grade) ± StdDev
PCa Urine	50	69.16 ± 6.89	10.82 ± 19.99	2.28 ± 1.32
BPH Urine	50	69 ± 9.93	4.85 ± 4.18	//

**Table 10:** List of DE RNAs in PCa selected by literature mining.

RNA Type	ID	Biological Sample	Expression Status	References
lncRNA	PCAT18	Plasma	Upregulated (positive correlation between lncRNA level and disease stage)	[38]
	MALAT1	Urine	Upregulated	[39]
	SChLAP1	Urine, plasma exosomes	Upregulated	[40]
	PCA3	Urine (whole urine and pellet), urine exosomes	Upregulated	[41,42]
	TP53COR1	Urine exosomes	Upregulated	[43,44]
	SAP30L-AS1a	Plasma exosomes	Downregulated	[44]
mRNA	TMPRSS2:ERG	Whole urines	Upregulated	[45,46]
	HOXC6	Pellet from urine	Upregulated	[47,48]
	TDRD1	Pellet from urine	Upregulated	[48]
	DLX1	Pellet from urine	Upregulated	[48,49]
	KLK3	Whole blood, whole urine	Upregulated	[42,49]
miRNA	miR-27b-3p	Urine, plasma, and tissue samples	Up- and Downregulated	[50]
	miR-30a-3p	Urine	Upregulated	[51]
	miR-30a-5p	Urine	Upregulated	[51]
	miR-30b-5p	Urine	Upregulated	[51]
	miR-30c-5p	Urine	Upregulated	[51]
	miR-107	Urine, serum	Upregulated	[52]
	miR-125b-5p	Urine	Upregulated	[51]
	miR-574-3p	Urine	Upregulated	[52]

**Table 11:** PCR primers for PCa-related long RNAs selected by literature data mining and the housekeeping gene.

RNA Type	Gene	Forward Primer	Reverse Primer	Product Length
**mRNA**	DLX1	CGGCCTCTTTGGGACTCAC	CAGCTTCTTGAACTTGGATCGC	74
	ERG	AAGTAGCCGCCTTGCAAAT	CAGCTGGAGTTGGAGCTGT	90
	HOXC6	CCCTGGATGCAGCGAATGA	GGTACCGCGAGTAGATCTGG	85
	KLK3	GAGCACCCCTATCAACCCCCTATT	AGCAACCCTGGACCTCACACCTAA	119
	TDRD1	GCGGTCCTCTTGCCTCTTTC	CATCTTACAAGGAGGTGCTTCCA	93
**Fusion mRNA**	TMPRSS2:ERG	CAGGAGGCGGAGGCGGA	GGCGTTGTAGCTGGGGGTGAG	*
**lncRNA**	MALAT1	TAGCTTGGATCCTTGTGGGC	AACCCACCAAAGACCTCGAC	73
	PCA3	TGGCACTATGAGCTGCCAAT	CCCCAGCTGAGACCTAATGC	247
	PCAT18	AGGAGACAGGCCCCAGATTT	TGAAGTGCTGGGACAACGTA	107
	SCHLAP1	AGAACCCACCAGTTCTGGACA	GGTGAAAGTGCCTTATACAGGTTGA	100
	TP53COR1	GGGTGGCTCACTCTTCTGGC	TGGCCTTGCCCGGGCTTGTC	100
	SAP30L-AS1	TGAATGGGCTCACCTGTTCC	AGGTCCGGAAGGGAGACTTT	159
**Housekeeping**	GAPDH	TGCACCACCAACTGCTTAGC	GGCATGGACTGTGGTCATGAG	87

* The transcript derived from the transmembrane serine protease 2:ETS transcription factor ERG (TMPRSS2:ERG) fusion gene does not exhibit an amplicon of standard length. Various isoforms have been described, each displaying specific breakpoints within the two genes, which vary from patient to patient. The predominant fusion form identified, as originally proposed by Tomlins et al. [53], involved the fusion of exon 1 of TMPRSS2 with exon 4 of ERG (T1/E4). In light of this information, the primer pair utilized in this study corresponds to that reported in the aforementioned work.

## Data Availability

Data are contained within the article or supplementary material.

## Supplementary Materials

The following supporting information can be downloaded at: https://www.mdpi.com/article/10.3390/ijms251810079/s1, Figures S1 and S2; Tables S1 and S2.
