# Supplementary material for: Extracellular RNAs from Whole Urine to Distinguish Prostate Cancer from Benign Prostatic Hyperplasia
Source: Int J Mol Sci. 2024 Sep 19;25(18):10079. doi: 10.3390/ijms251810079 (PMC11432375; doi:10.3390/ijms251810079)
Supplement: Supplementary file 1 [file ijms-25-10079-s001.zip › ijms-3175838-supplementary.pdf]

International Journal of
Molecular Sciences

MDPI

Supplementary Materials

Table S1. Total RNA yields extracted from urine fractions. Total RNA yields extracted from whole urine and urine fractions obtained after centrifugation (pellet and supernatant) both in prostate cancer (PCa) and benign prostatic hyperplasia (BPH) samples (8 vs 8).

Sample	PCa Mean RNA Yield	BPH Mean RNA Yield
	(ng) ± StdDev	(ng) ± StdDev
**Whole Urine**	130 ± 56.69	2024.38 ± 1797.20
**Pellet from Urine**	100.05 ± 46.24	1553.03 ± 1400.71
**Centrifuged Urine (supernatant)**	31.58 ± 11.42	519.81 ± 505.96

Table S2. Comparison of commercial extraction kits. A test set of 5 urine samples from healthy donors was used to evaluate the RNA extraction kits. The starting volume was the same for all extractions. The manufacturer protocol was used for the extraction. The table presents the mean RNA yields obtained with each kit. Additionally, it reports the mean Ct values for miR-16-5p and glyceraldehyde 3-phosphate dehydrogenase (GAPDH), which were used to assess extraction efficiency. For each PCR reaction, 10 ng of total RNA was used as input for miR-16-5p, and 50 ng for GAPDH following the protocols described in the Materials and Methods section.

Extraction Kit	Mean RNA Yield	Mean miR-16-5p Ct	Mean GAPDH Ct
	**(ng) ± StdDev**	**Ct ± StdDev**	**Ct ± StdDev**
**miRNeasy Kit (QIAGEN)**	968 ± 255.56	24.80 ± 0.96	26.47 ± 1.71
**miRNeasy Serum/Plasma Kit (QIAGEN)**	1081 ± 302.56	26.67 ± 0.85	27.80 ± 1.46
**Plasma/Serum RNA Purification Kit (NORGEN)**	920 ± 217.12	28.68 ± 1.33	28.67 ± 0.92
**Urine Cell-Free Circulating RNA Purification Kit (NORGEN)**	1688 ± 469.26	21.81 ± 0.57	25.28 ± 1.35

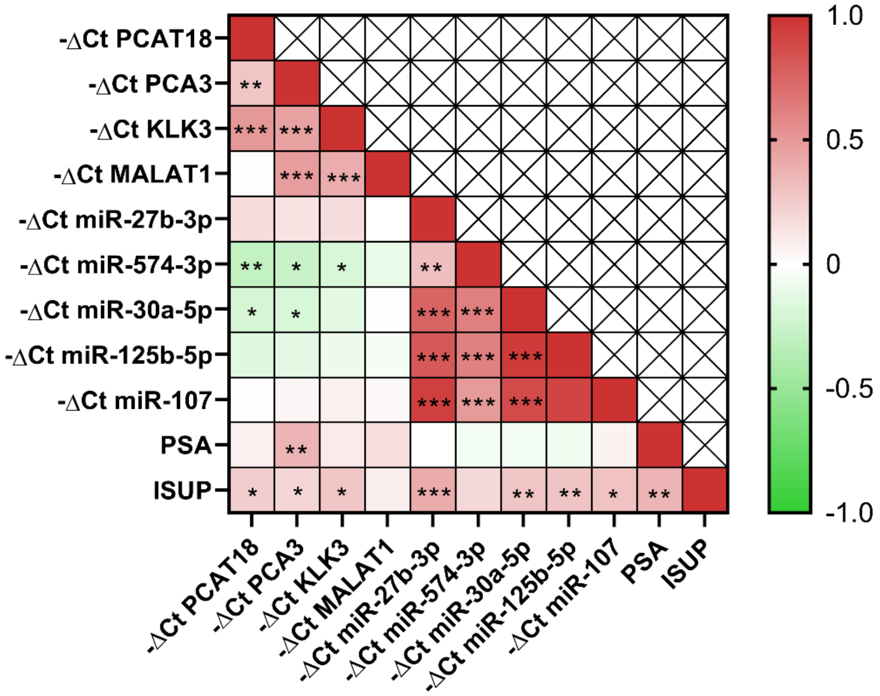

Figure S1. Correlation between differentially expressed RNAs in Urine and PSA and ISUP by using data from Real-Time PCR. Heatmap showing correlations between the expression of RNA transcripts and clinicopathological data (prostate-specific antigen [PSA] value and International Society of Urological Pathology grade [ISUP]); correlation coefficients grade showed in our cohort (PCa = 50 vs BPH = 50) a positive and significant correlation between ISUP grade and the expression levels of most of the transcripts examined (prostate cancer associated transcript 18 [PCAT18], prostate cancer-associated 3 [PCA3], miR-27b-3p, miR-125b-5p, miR-107) (Figure S1). PSA values were positively correlated to the expression of PCA3. The scale bar shows *r-values* calculated through Spearman correlation tests, ranging from green (negative correlation) to red (positive correlation). Statistical significance is represented by the number of asterisks (*= *p-value* <0.05; ** = *p-value* <0.005; *** = *p-value* <0.0005).

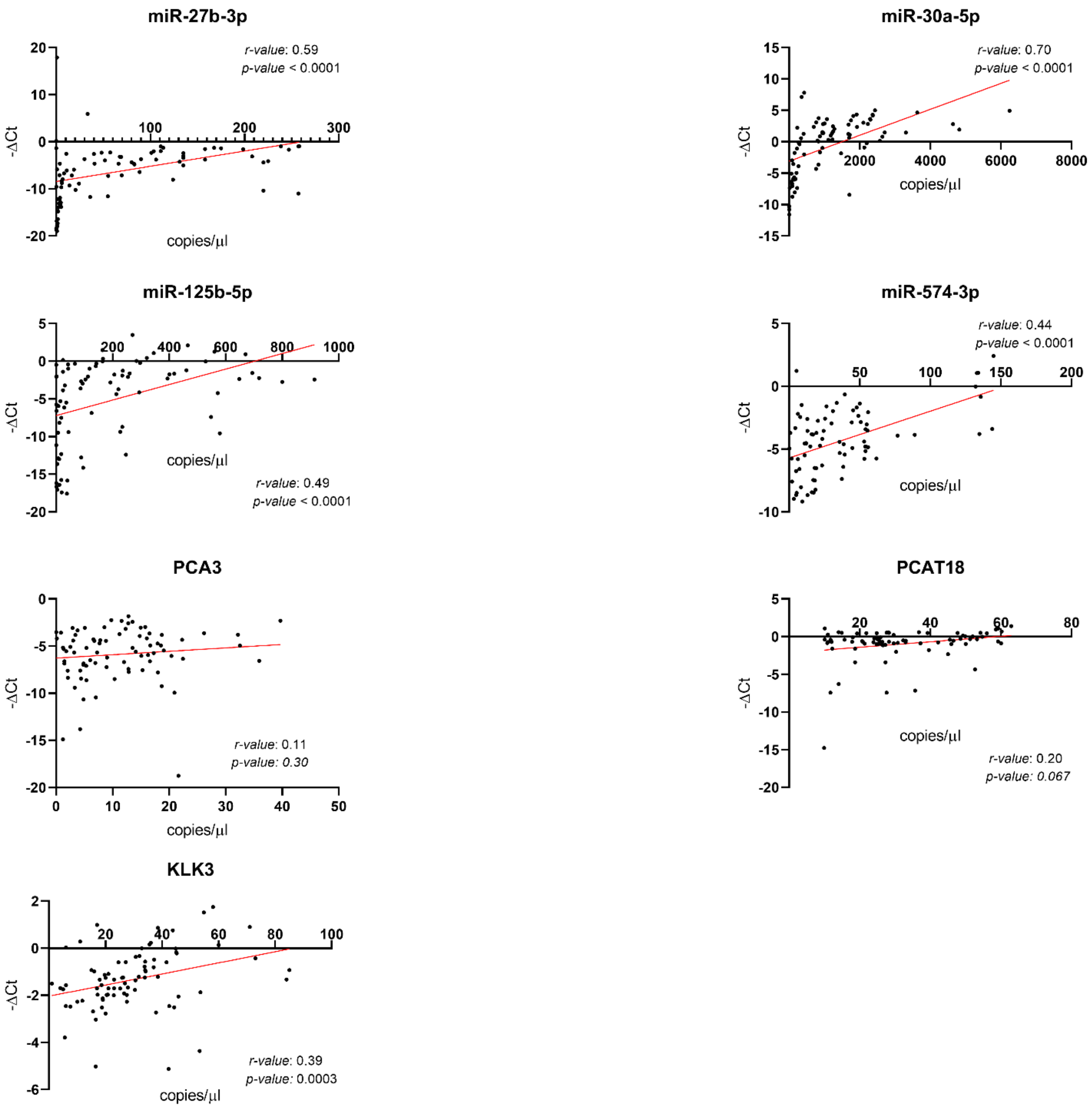

Figure S2. Correlation between Real-Time PCR and droplet digital (ddPCR) data. The graphs show correlations between Real-Time PCR data expressed as -ΔCt and ddPCR data expressed as copies/µL, for the transcript analyzed with each of the two techniques. R-values were calculated through Spearman correlation test, and statistical significance is represented by *p-value*.
